# IFNβ drives ferroptosis through elevating TRIM22 and promotes the cytotoxicity of RSL3

**DOI:** 10.3389/fimmu.2025.1535554

**Published:** 2025-02-05

**Authors:** Huiyue Dong, Ling Zhu, Jingjing Sun, Qiuyan Chen, Pengyang Liu, Wei Zhang, Huajing Zeng, Rong Lin, Zongyang Yu, Jun Lu

**Affiliations:** ^1^ Fujian Provincial Key Laboratory of Transplant Biology, Dongfang Hospital (the 900th Hospital of Joint Logistic Support Force), Xiamen University, Fuzhou, China; ^2^ Laboratory of Basic Medicine, Fuzong Clinical Medical College of Fujian Medical University, Fuzhou, China; ^3^ Clinical Laboratory, Wuhan Children’s Hospital, Tongji Medical College Huazhong University of Science and Technology, Wuhan, China; ^4^ Fuzong Teaching Hospital, Fujian University of Traditional Chinese Medicine, Fuzhou, China; ^5^ Department of Pulmonary and Critical Care Medicine, Fuzong Clinical Medical College of Fujian Medical University, Fuzhou, China; ^6^ Organ transplant institute, Dongfang Hospital, Xiamen University, Fuzhou, China

**Keywords:** IFNβ, ferroptosis, cGAS-STING, TRIM22, RSL3

## Abstract

**Background:**

Cyclic GMP-AMP synthase (cGAS)-stimulator-of-interferon genes (STING) pathway is a cytosolic DNA sensor system. The production of this pathway, interferon-β (IFNβ), could suppress the growth of tumor cells, yet it is unclear whether ferroptosis is involved in IFNβ-induced cell death.

**Methods:**

The effects of IFNβ on ferroptosis were analyzed in HT1080, 4T1, HCT116 and 786-O cells. HT1080 and 4T1 cells treated with IFNβ were subjected to RNA-Seq analysis. STAT1, STAT3, TRIM21, and TRIM22 were silenced by siRNAs to examine their effects on IFNβ-induced ferroptosis. The cGAS-STING signaling pathway-activated mice were used to evaluate the effects of IFNβ on ferroptosis *in vivo*. HT1080 cells, three-dimensional (3D) spheroids, and the xenograft mouse models were treated with IFNβ, RSL3, or IFNβ combination with RSL3 to analyze whether IFNβ enhances RSL3-induced ferroptosis.

**Results:**

Here, we found that IFNβ could promote intracellular Fe^2+^ and lipid peroxidation levels, and decrease GSH levels in tumor cells. RNA sequencing data revealed that IFNβ induced a transcriptomic disturbance in ferroptosis-related genes. Knockdown of tripartite motif-containing 22 (TRIM22) suppressed the levels of intracellular Fe^2+^ and lipid ROS. It also reduced heme oxygenase (HMOX1) protein levels and increased ferroptosis suppressor protein 1 (FSP1) levels in HT1080 cells treated with IFNβ. Furthermore, our results illustrated that IFNβ enhanced the RAS-selective lethal 3 (RSL3)-induced ferroptosis and the inhibitory effect of RSL3 on GPX4. Meanwhile, compared to the groups treated with either IFNβ or RSL3 alone, the combination treatment of IFNβ and RSL3 significantly inhibited the growth of HT1080 three-dimensional (3D) spheroids and tumor in a mouse xenograft model.

**Conclusions:**

Our work reveals a role for IFNβ in promoting ferroptosis and provides evidence that IFNβ could be used with RSL3 to increase cytotoxic effects in tumor cells.

## 1 Introduction

Ferroptosis is a recently discovered form of programmed cell death characterized by iron-dependent lipid peroxidation (1). Several small-molecule ferroptosis inducers promote cell death by depleting intracellular glutathione (GSH), directly targeting GPX4 activity or increasing intracellular Fe^2+^, such as RSL3, which inhibits GPX4 activity and leads to the accumulation of intracellular lipid peroxide (2). Tumor cells are susceptible to ferroptosis inducers, as they have higher levels of Fe^2+^, increasing the promise of these inducers to be applied in cancer therapy (3).

The cGAS-STING signaling pathway detects intra-cytoplasmic DNA in mammalian cells to trigger an innate immune reaction (4). Within this pathway, double-stranded DNA (dsDNA) activates the catalytic activity of cGAS. It induces the production of cyclic guanosine monophosphate-adenosine monophosphate (cGAMP), a second messenger molecule and agonist of STING (5). cGAMP activates the endoplasmic reticulum transmembrane protein STING, then recruits TANK-binding kinase 1 (TBK1) to activate interferon regulatory factor 3 (IRF3) via phosphorylation. Activated IRF3 enters the nucleus to promote IFNβ expression (6). STING agonists or cGAMP have potent anti-tumor effects *in vivo* (7). However, whether the cGAS-STING signaling pathway acts against tumors by mechanisms other than enhancing natural immunity remains to be established.

IFNβ is the production of the cGAS-STING pathway (8). Besides antiviral activity, IFNβ could also reduce tumor growth by inducing anti-proliferative and apoptotic effects and promoting systemic immunity against the tumor targets (9). However, the role of ferroptosis in IFNβ-mediated cell death and tumor suppression has not been identified. The aims of this study were: 1) to verify whether IFNβ promotes ferroptosis in tumor cell lines; 2) to investigate which proteins play essential roles in IFNβ-mediated ferroptosis; 3) to observe whether IFNβ can synergize with classical ferroptosis inducer RSL3 to exert anti-tumor effects.

## 2 Materials and methods

### 2.1 Cell culture and treatment

HT1080 (human fibrosarcoma cell line) (cat number: CL-0117), 4T1 (mouse mammary tumor cell line) (cat number: CL-0007), HCT116 (human colorectal carcinoma cell line) (cat number: CL-0096), and 786-O (human clear cell renal carcinoma cell line) (cat number: CL-0010) were obtained from Procell Co., Ltd. (Wuhan, China). HT1080 and HCT116 were cultured in Dulbecco’s Modified Eagle Medium (DMEM, high glucose) containing 10% fetal bovine serum, 100 units/mL penicillin, and 100 µg/mL streptomycin (Thermo Fisher Scientific). 4T1 and 786-O were cultured in Roswell Park Memorial Institute (RPMI) 1640 medium containing 10% fetal bovine serum, 100 units/mL penicillin, and 100 µg/mL streptomycin (Thermo Fisher Scientific). All the cells were cultured at 37°C in a humidified incubator with 5% CO_2_. To induce ferroptosis, 4T1 cells were treated with mouse IFNβ, while other cells were treated with human IFNβ at a concentration of 20 ng/mL (Sinobiological, China) for 48 hours. In the combination treatment, RSL3 (Med Chem Express, cat number: HY-100218A) (0.5 μM) was added to the cultures with 20 ng/mL IFNβ.

### 2.2 Cell viability assay

Cell viability was detected using a cell counting kit (CCK8) (MedChemExpress, cat number: HY-K0301). In brief, cells were seeded into 96-well cell plates and treated with IFNβ or other compounds the next day. After 48 h, CCK8 (10 μL/well) was added, and the cells were incubated for one hour. The optical density of each well was measured by a spectrophotometer (Multiskan GO, Thermo Fisher Scientific) at a wavelength of 450 nm. Six repeat wells were set for each group. To evaluate the rescue effect of ferroptosis inhibitor, liproxstatin-1 (10 μM) (MedChemExpress, cat number: HY-12726) was added to the cultures with 20 ng/mL IFNβ.

### 2.3 Lipid peroxide level assay

Cells were seeded and treated with IFNβ (20 ng/mL) or combined with RSL3 (0.5 μM) the next day. Lipid peroxides were detected by C11-BODIPY 581/591 (cat number: D3861, Thermo Fisher Scientific) 24 h later. The cells were incubated with a C11-BODIPY medium (10 μM) at 37°C for 30 min. FACScan (Becton Dickinson; FACSAriaII) measured the C11-BODIPY fluorescence intensity. The results were analyzed with FlowJo V10 software.

### 2.4 Intracellular iron assay

Intracellular Fe^2+^ levels were assessed using a FerroOrange (Fe^2+^ indicator) kit (cat number: F374, Dojindo, Japan). Cells were seeded in 6-well plates and treated with IFNβ (20 ng/mL), RSL3 (0.5 μM), or their combination the next day. FerroOrange (1 μM), dispersed in serum-free medium, was added to the cells 24 h later, and the cells were incubated for 30 min at room temperature and protected from light. The absorbance was subsequently observed by a fluorescence microscope or measured by flow cytometry. Fluorescence microscopy images were quantitatively analyzed using ImageJ software, examining six regions per sample. The images were converted to RGB format, and thresholding was applied to distinguish positively stained cells from the total cell population. Using ImageJ’s “Analyze” menu and “Measure” function, we obtained AOD (% Area) values. The proportion of positive cells was determined by comparing AOD values between the experimental and control groups.

### 2.5 Viability staining

Live cells were stained with calcein AM showing green fluorescence, while dead cells were stained with propidium iodide (PI) showing red fluorescence. Following the kit instructions (cat number: C2015M, Beyotime, China), 3D spheroids were stained and photographed under a fluorescence microscope after 30 minutes. Fluorescence quantitative analysis of microscope images was performed using ImageJ software to analyze positively stained cells in 6 spheroids. The images were converted to RGB format, and thresholding was applied to distinguish positively stained cells from the total cell population. Using ImageJ’s “Analyze” menu and “Measure” function, we obtained AOD (% Area) values. The proportion of positive cells was determined by comparing AOD values between the experimental and control groups.

### 2.6 GSH assay

According to the manufacturer’s instructions, individual levels of GSH were measured using a GSH assay kit (Colorimetric) (cat number: ab239727). The optical density of each well at a wavelength of 450 nm was measured by a spectrophotometer (Multiskan GO, Thermo Scientific).

### 2.7 Cell transfection

For knockdown of STAT1, STAT3, TRIM21 and TRIM22, siRNAs or a non-targeting control siRNA were purchased from HippoBio (Huzhou, China). The selected siRNA sequences are listed in **Supplementary Table S1**. The cells were transfected with Lipofectamine RNAiMAX (cat number: 13778075, Thermo Fisher Scientific, Inc.) using 3 μL (30 pmol) of siRNA per well in a 6-well plate. Transfection was performed when the cell confluence reached 60-80% following the manufacturer’s instructions.

### 2.8 Reverse-transcription quantitative polymerase chain reaction

Total cellular RNA was extracted using Total RNA Kit II (cat number: R6934, Omega Bio-Tek, Inc., Norcross, GA, USA). The RNA samples were treated with DNase. After RNA quantification, 3 μg of total RNA was reverse transcribed into cDNA using the RevertAid First Strand cDNA Synthesis kit, following MIQE guidelines (10). PowerUp SYBR Green mix (cat number: A25741, Applied Biosystems, Thermo Fisher Scientific, Inc.) was used for qPCR. PCR was carried out for 40 cycles under the following conditions: 30 seconds at 94°C and 60 seconds at 58°C. Data were normalized to ACTB. The relative expression levels of genes were calculated according to 2-ΔΔCT methods (11). Primers used in the qPCR assays are shown in **Supplementary Table S2**.

### 2.9 RNA sequencing analysis

For RNA sequencing, total RNA was extracted from HT1080 and 4T1 cells treated with or without 20 ng/mL mouse IFNβ for 24 h. The enriched mRNA is then converted into complementary DNA (cDNA) through reverse transcription. Then, the cDNA is fragmented and adapters are ligated to its ends for library preparation. Following library preparation, fragment distribution and quantification performed to ensure optimal library quality. The library is sequenced using the Illumina Novaseq 6000 sequencing platform. Raw sequencing reads were demultiplexed using bcl2fastq software. Reads were aligned to the human or mouse reference genome. Differential expression analysis is performed using the DESeq2 library in the R statistical environment to identify genes with significant expression changes between IFNβ treated and untreated HT1080 or 4T1 cells. The enrichment analysis of differentially expressed genes (DEGs) was analyzed by Metascape (https://metascape.org).

### 2.10 Western blotting

The concentrations of proteins were quantified by a bicinchoninic acid (BCA) kit (Thermo Fisher Scientific). Cellular proteins (15 μg/sample) were separated by sodium dodecyl sulfate-polyacrylamide gel electrophoresis (SDS-PAGE) and transferred to polyvinylidene fluoride (PVDF) membranes (Millipore, Billerica, MA, USA). PVDF membranes were blocked in a blocking solution (in PBS with 0.1% Tween 20 and 2% BSA) for 1 h and then incubated with primary antibodies at 4°C overnight. The membranes were washed and incubated with horseradish peroxidase (HRP)-labeled antibody at 37°C for 1 h. Immunostaining was developed using the enhanced chemiluminescence detection system (WesternBright ECL, Advansta) and recorded by Alliance Q9 Advanced (UVItec, ltd. Cambridge). All primary antibodies used in this study are listed in **Supplementary Table S3**.

### 2.11 Animal experiments

All mouse procedures in this study were approved by the Animal Care and Use Committee of the 900th hospital (approval number: 2021-025) on December 31, 2021. These experiments have been performed in accordance with the Basel Declaration (http://www.basel-declaration.org/basel-declaration/). To stimulate the cGAS-STING pathway in mouse models, C57 mice were injected with cGAMP (MedChemExpress, Shanghai, China) from the tail vein. Twelve male C57 mice aged 8 weeks were randomly divided into two groups (n = 6 per group): 1) vehicle control; 2) cGAMP-infused group (6 mg/kg). The mice received cGAMP every other day for four times.

Six-week-old male BALB/c nude mice were provided by the Shanghai Laboratory Animal Center (Shanghai, China). HT1080 cells (5 × 10^5^) suspended in 100 μL PBS/Matrigel (1:1) were injected into the hind flank of the mice. Then, the mice were randomly divided into four groups (n = 5 per group): 1) vehicle control; 2) IFNβ (mouse IFNβ, 100 ng per mouse, administered by intra-tumor injection); 3) RSL3 (2.5 mg/kg, administered by intraperitoneal injection); 4) or IFNβ co-treatment with RSL3. The mice received drugs every other day for seven consecutive times. At the endpoints, tumor tissues were removed to analyze intracellular Fe^2+^, lipid peroxidation, and GSH.

### 2.12 Immunohistochemical staining

Tumor tissues were fixed, embedded into the paraffin, and sectioned in standard procedure. After antigen retrieval and blocking, the slides were incubated with Ki67 antibody (1:800; Cell Signaling Technology) at 4°C overnight. Slides were washed and incubated with a secondary antibody (horseradish-peroxidase–conjugated, 1:500; ZSGB-BIO, Beijing, China). After DAB staining, the slides were counterstained with hematoxylin. Images were captured under a microscope (Olympus, BX43). Ki67-positive nuclei were quantified in five regions, with approximately 2000 cells analyzed per image using ImageJ. The images were converted to RGB stack format, and a threshold was set to distinguish between total nuclei and the positively stained nuclei. The measurements were obtained by selecting the “Analyze” menu in ImageJ and choosing the “Measure” option, which provided the AOD (%Area) value. The AOD value of Ki67-positive nuclei was then compared to the total nuclei AOD value to calculate the percentage of Ki67-positive cells.

### 2.13 Statistical processing

The data obtained are presented as mean ± S.D. Statistical significance was measured by oneway ANOVA (for more than two comparisons) and Student’s t-test (comparison of two groups) (*P* values). *, *P* < 0.05; **, *P* < 0.01; n.s., non-significant. All the statistical graphs were performed using GraphPad Prism software version 8.0. The results of *in vitro* experiments were collected from at least three independent biological replicates.

## 3 Results

### 3.1 IFNβ induces tumor cell death partially through ferroptosis

**Figure 1:**
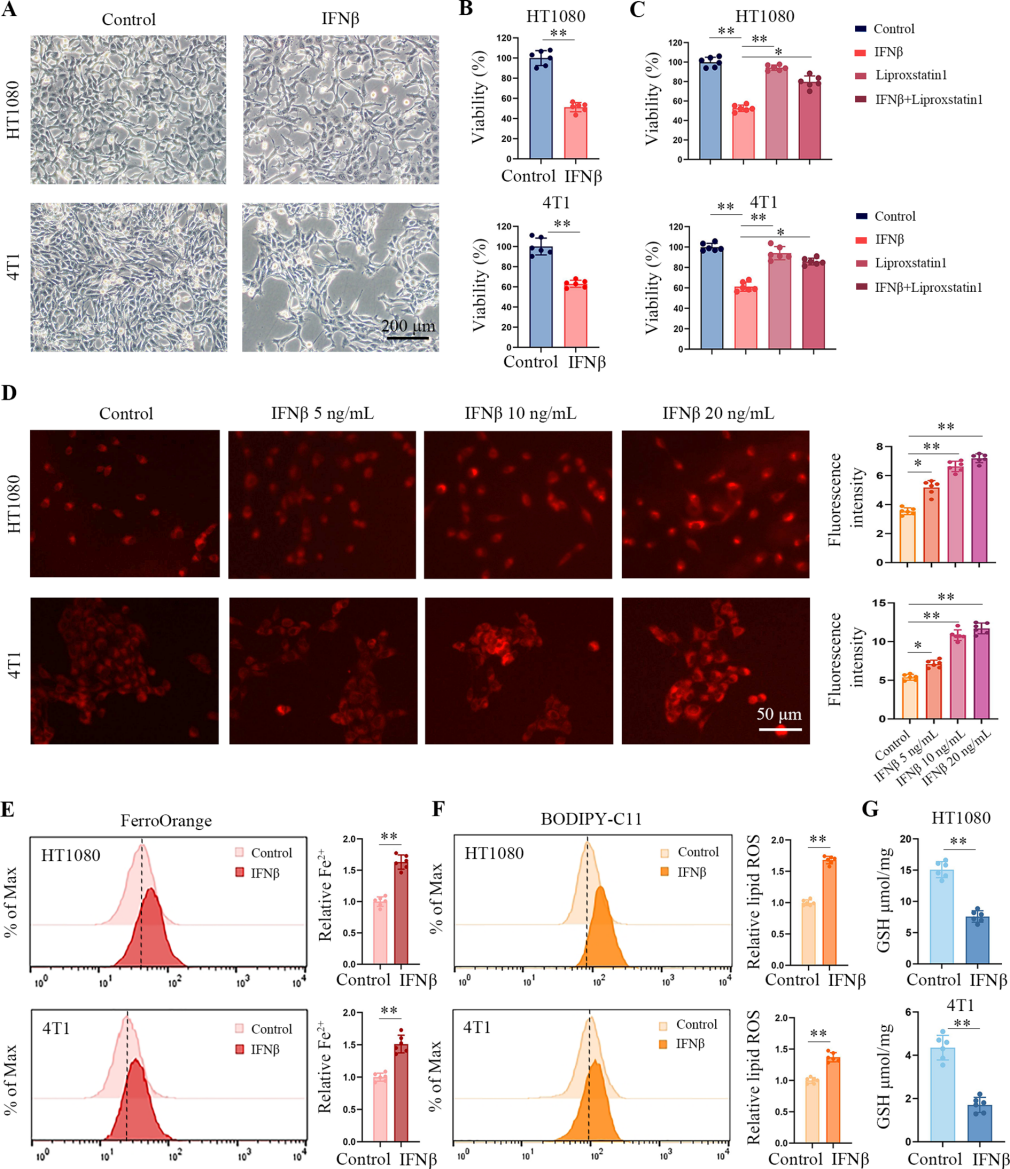
IFNβ treatment induces ferroptosis in tumor cells. **(A)** Representative images by inverted light microscopy show morphology changes in HT1080 and 4T1 cells treated with IFNβ (20 ng/mL) for 48 h. Scale bar: 200 μm. One representative experiment of three independent experiments is shown. **(B)** The CCK-8 assay determined the inhibitory effects of IFNβ (20 ng/mL). Data indicated as mean ± S.D. (n = 6 replicates). One representative experiment of three independent experiments is shown. **(C)** CCK-8 assay showing the response of HT1080 and 4T1 cell lines to IFNβ (20 ng/mL) with or without liproxstatin-1 (10 μM) for 48 h. Data indicated as mean ± S.D. (n = 6 replicates). One representative experiment of three independent experiments is shown. **(D)** Representative images show intracellular Fe^2+^ levels in HCT1080 and 4T1 cells treated with various concentrations of IFNβ (0 ng/mL, 5 ng/mL, 10 ng/mL, and 20 ng/mL) for 24 h. Scale bar: 50 μm. Data indicated as mean ± S.D. (n = 6 experiments). **(E, F)** Flow cytometric analysis of intracellular Fe^2+^ and lipid peroxydation levels in HCT1080 and 4T1 cells treated with or without 20 ng/mL IFNβ for 24 h. Data indicated as mean ± S.D. (n = 6 experiments). **(G)** GSH levels of HCT1080 and 4T1 cells primed with or without 20 ng/mL IFNβ for 24 h. Data indicated as mean ± S.D. (n = 6 experiments). Statistical significance relative to mock conditions are indicated as *P < 0.05, **P < 0.01.

Human HT1080 fibrosarcoma cells and murine 4T1 breast tumor cells were treated with IFNβ for 48 h. We observed IFNβ increased cell death in both cell lines (**Figures 1A, B**). **Figure 1C** shows that the ferroptosis inhibitor liproxstatin-1 reduced the cytotoxic effect of IFNβ in HT1080 and 4T1 cells. Then, we quantified the iron level in HT1080 and 4T1 cells using a Fe^2+^ iron probe named FerroOrange. Consistent with our expectation, IFNβ increased the intracellular Fe^2+^ concentrations within a dose-dependent manner (**Figure 1D**). Flow cytometric results also demonstrated an increment of Fe^2+^ in IFNβ-treated HT1080 and 4T1 cells (**Figure 1E**). Moreover, IFNβ treatment enhanced lipid peroxidation as assessed by the lipid peroxydation sensor BODIPY-C11 (**Figures 1F**, **Supplementary Figure S1**), but decreased glutathione (GSH) levels to 40% in HT1080 and 4T1 cells (**Figure 1G**). Human renal cell carcinoma cell line 786-O and colon cancer cell line HCT116 cells were treated with IFNβ for 48 h. IFNβ treatment increased cell death, intracellular Fe^2+^ and lipid peroxidation levels (**Supplementary Figure S2**). Transmission electron microscopy analysis revealed that IFNβ significantly induced mitochondrial cristae disappearance and outer membrane rupture in 4T1 and HT1080 cells (**Supplementary Figure S3**). Collectively, these findings suggested that IFNβ induced ferroptosis in tumor cells.

### 3.2 IFNβ treatment stimulates ferroptosis regulators

**Figure 2:**
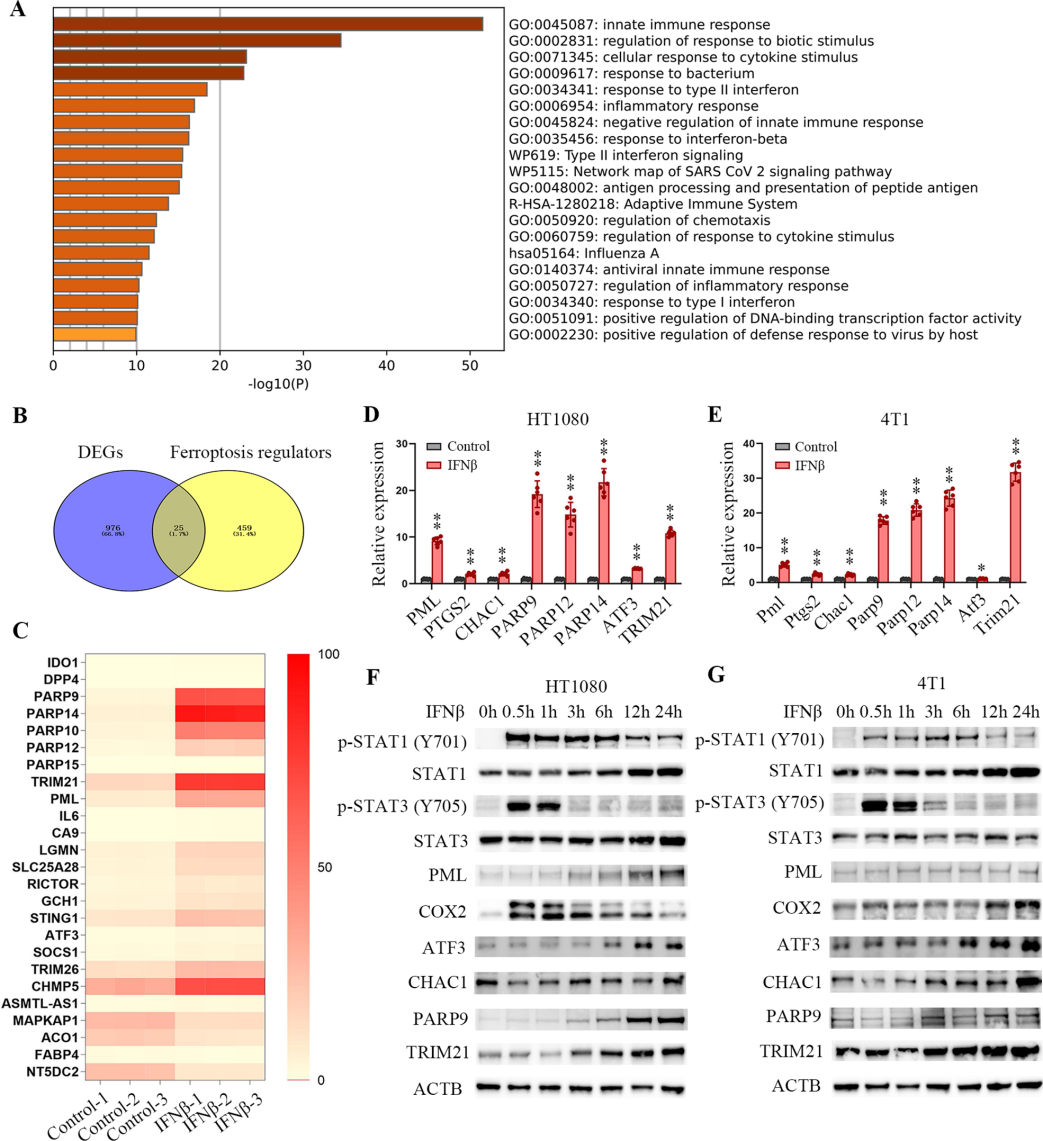
IFNβ treatment changes ferroptosis-related gene expression. **(A)** Bar graph of functional enrichment analysis by Metascape. **(B)** Venn diagrams showing the differentially expressed genes (DEGs) that overlapped among ferroptosis regulators. **(C)** Heatmap of ferroptosis regulators detected by the RNA-seq. The heatmap shows a fold-change of ferroptosis genes that range from zero to 100 between IFNβ untreated and treated HT1080 cells (n = 3 samples). **(D, E)** mRNA levels of ferroptosis regulators normalized to ACTB and determined by RT-qPCR. Bar plots represent the fold-change of each gene relative to mRNA levels from IFNβ-untreated HT1080 or 4T1 cells. Data indicated as mean ± S.D. (n = 6 experiments). **(F, G)** Representative images of western blotting analysis show the protein levels of STAT1, STAT3, PML, COX2, ATF3, CHAC1, PARP9, TRIM21 and phosphorylation status of STAT1 and STAT3 in HT1080 and 4T1 cells treated with 20 ng/mL IFNβ for the indicated time points. Statistical significance relative to mock conditions is indicated as *P < 0.05 and **P < 0.01.

To further decipher the molecular mechanism that may account for IFNβ stimulation of ferroptosis, we performed whole-transcriptome sequence analysis (RNA-seq) to detect the gene expression changes after IFNβ treatment. IFNβ exposure caused significant alterations in gene expression compared to untreated HT1080 cells (**Figure 2A**). A set of ferroptosis-related genes was obtained from the FerrDb database (http://www.zhounan.org/ferrdb/current/). We identified 25 ferroptosis-related differentially expressed genes (DEGs) (**Figure 2B**, **Supplementary Table S4**), among which 21 were upregulated, four were downregulated in HT1080 cells (**Figure 2C**). Similar results were identified in 4T1 cell (**Supplementary Figure S4A**), IFNβ changed 18 ferroptosis-related genes (**Supplementary Figure S4B**, **Supplementary Table S5**), among which 16 were upregulated, two were downregulated (**Supplementary Figure S4C**). RT-qPCR assays proved that PML, PTGS2, CHAC1, PARP9, PARP12, PARP14, ATF3 and TRIM21 were upregulated in both IFNβ-treated HT1080 and 4T1 cells (**Figures 2D, E**). Western blot analysis demonstrated that IFNβ treatment led to rapid activation of signal transducer and activator of transcription 1 (STAT1) and STAT3 in both cell lines, as evidenced by enhanced phosphorylation levels within 30 minutes of exposure. Furthermore, IFNβ exposure resulted in upregulated protein expression of several key factors, including COX2, ATF3, CHAC1, PARP9, and TRIM21 (**Figures 2F, G**). These findings suggest that besides the immune response genes, IFNβ disturbed several ferroptosis-related gene expressions in tumor cells.

To investigate the ferroptosis effect induced by IFNβ *in vivo*, we treated the mice with cGAMP to stimulate the cGAS-STING pathway. ELISA analysis shows cGAMP induced the amount of IFNβ in serum (**Supplementary Figure S5A**). As the heart is sensitive to ferroptosis (12), we focused on cardiac tissues to investigate the influence of IFNβ. We found that cGAMP-injection promoted the expression level of Ifnb1 in the heart (**Supplementary Figure S5B**). The activation of STAT1/STAT3 pathways suggested that cardiac cells were attacked by IFNβ (**Supplementary Figure S5C**). Compared to non-injected mice control, cGAMP treatment enhanced intracellular Fe^2+^ and lipid peroxidation levels (**Supplementary Figures S5D, E**) and significantly decreased GSH levels in heart tissues (**Supplementary Figure S5F**). Moreover, cGAMP-infusion induced overexpression of ferroptosis-related genes in the heart (**Supplementary Figure S5G**). These results supported the notion that systemic activation of the cGAS-STING pathway could induce ferroptosis.

### 3.3 Inhibition of TRIM22 mitigates IFNβ-mediated ferroptosis

**Figure 3:**
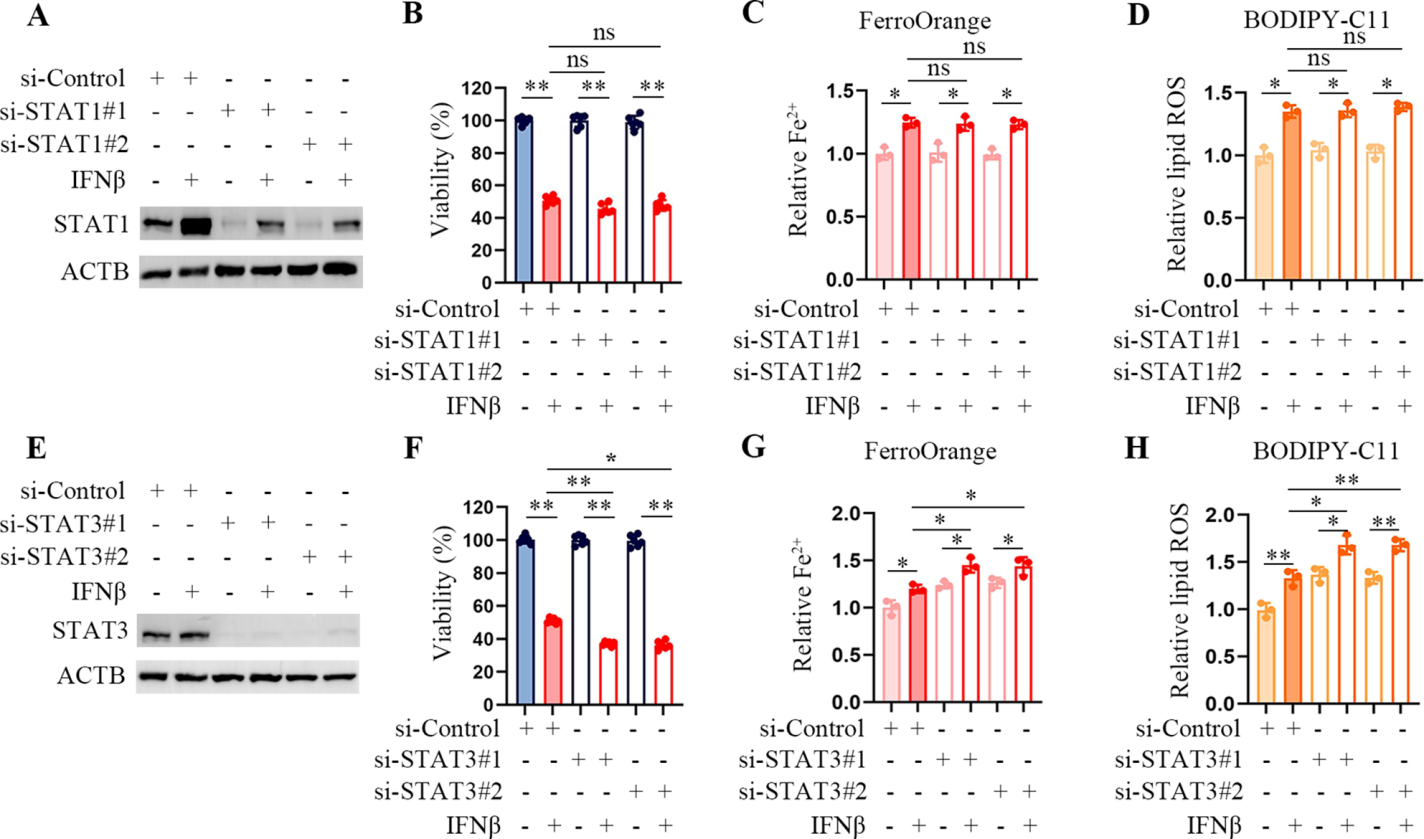
STAT3 downregulation enhances IFNβ-mediated ferroptosis. **(A)** Representative images of western blotting analysis of STAT1 in IFNβ untreated and treated HT1080 cells transfected with control or STAT1 siRNAs. **(B)** CCK-8 assay of HT1080 cells transfected with control or STAT1 siRNAs in the presence or absence of 20 ng/mL IFNβ for 48 h. Data indicated as mean ± S.D. (n = 6 replicates). One representative experiment of three independent experiments is shown. **(C, D)** Flow cytometric analysis of intracellular Fe2^+^ and lipid ROS levels in HCT1080 cells transfected with control or STAT1 siRNAs followed by treatment with or without IFNβ for 24 h. Data indicated as mean ± S.D. (n = 3 experiments). **(E)** Representative images of western blotting analysis of STAT3 in IFNβ untreated and treated HT1080 cells which transfected with control or STAT1 siRNAs. **(F)** CCK-8 assay of HT1080 cells transfected with control or STAT3 siRNAs in the presence or absence of 20 ng/mL IFNβ for 48 h. Data indicated as mean ± S.D. (n = 6 replicates). One representative experiment of three independent experiments is shown. **(G, H)** Flow cytometric analysis of intracellular Fe^2+^ and lipid peroxydation levels in HCT1080 cells transfected with control or STAT3 siRNAs followed by treatment with or without IFNβ for 24 h. Data indicated as mean ± S.D. (n = 3 experiments). Statistical significance relative to mock conditions is indicated as ns, non-significant, *P < 0.05, **P < 0.01.

Signal transducers and activators of transcriptions (STATs) play a crucial role in the cellular response to IFNβ. Recent studies have described how STAT1 and STAT3 participate in ferroptosis, but their roles need to be more consistent (13–15). We observed that STAT1 knockdown did not affect Fe^2+^ levels and lipid peroxydation accumulation in IFNβ-treated HT1080 cells (**Figures 3A–D**). STAT3 inhibition upregulated the level of intracellular Fe^2+^ and the product of lipid peroxidation and reduced cell viability in HT1080 cells (**Figures 3E–H**). These results suggested that inhibition of STAT1 did not affect IFNβ-mediated ferroptosis, and inhibition of STAT3 even enhanced ferroptosis in HT1080 cells.

**Figure 4:**
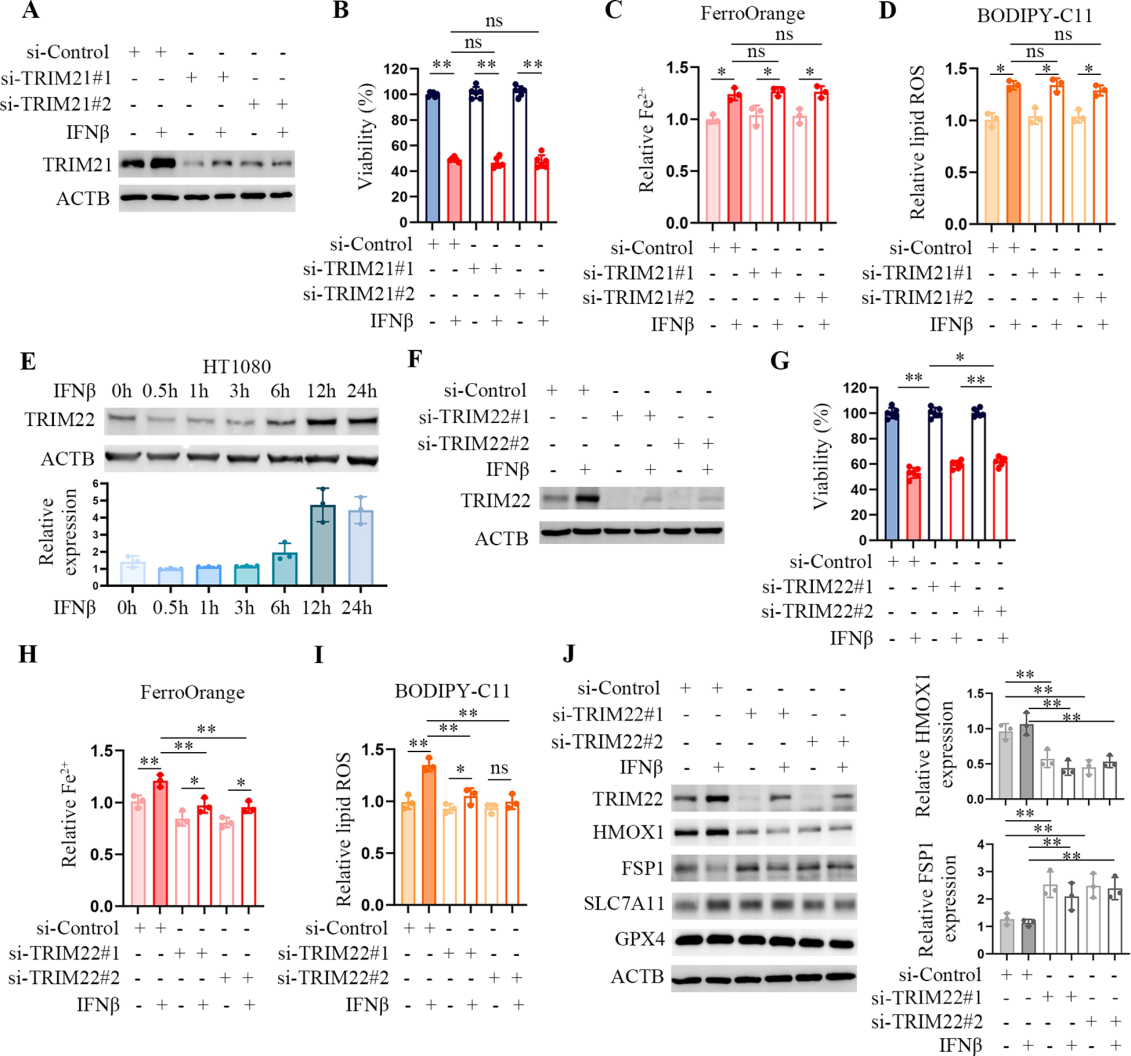
TRIM22 downregulation mitigates IFNβ-mediated ferroptosis. **(A)** Representative images of western blotting analysis of TRIM21 in IFNβ-treated or untreated HT1080 cells transfected with control or TRIM21 siRNAs. **(B)** CCK-8 assay of HT1080 cells transfected with control or TRIM21 siRNAs in the presence or absence of 20 ng/mL IFNβ for 48 h. Data indicated as mean ± S.D. (n = 6 replicates). One representative experiment of three independent experiments is shown. **(C, D)** Flow cytometric analysis of intracellular Fe^2+^ and lipid ROS levels in HCT1080 cells transfected with control or TRIM21 siRNAs followed by treatment with or without IFNβ for 24 h. Data indicated as mean ± S.D. (n = 3 experiments). **(E)** Representative images of western blotting analysis showing the levels of TRIM22 in HT1080 cells treated with 20 ng/mL IFNβ for the indicated time points. **(F)** Representative images of western blotting analysis of TRIM22 in HT1080 cells transfected with control or TRIM22 siRNAs in the presence or absence of IFNβ for 24 h. **(G)** CCK-8 assay of HT1080 cells transfected with control or TRIM22 siRNAs in the presence or absence of 20 ng/mL IFNβ for 48 h. Data indicated as mean ± S.D. (n = 6 replicates). One representative experiment of three independent experiments is shown. **(H, I)** Flow cytometric analysis of intracellular Fe^2+^ and lipid ROS levels in HCT1080 cells transfected with control or TRIM22 siRNAs followed by treatment with or without IFNβ for 24 h. Data indicated as mean ± S.D. (n = 3 experiments). **(J)** Representative images of western blotting analysis showing the protein levels of TRIM22, HMOX1, FSP1, SLC7A11 and GPX4 in HCT1080 cells transfected with control or TRIM22 siRNAs followed by treatment with or without IFNβ for 24 h. Statistical significance relative to mock conditions is indicated as ns, non-significant, *P < 0.05, **P < 0.01.

Tripartite motif-containing (TRIM) family proteins play a crucial role in regulating various substrates and signaling pathways. Previous studies show that TRIM21 is involved in ferroptosis (16, 17). Hence, we supposed TRIM21 activity was required for IFNβ-induced ferroptosis. However, TRIM21 deficiency did not affect Fe^2+^ levels or lipid peroxydation accumulation in IFNβ-treated HT1080 cells (**Figures 4A–D**). TRIM22 is another member of the IFNβ-induced protein and a transcriptional target of TP53 (18). We found that the expression of TRIM22 was enhanced in IFNβ-treated HT1080 cells (**Figure 4E**). TRIM22 was not detected in 4T1 cells because TRIM22 is not expressed in mice. TRIM22 depletion reduced intracellular Fe^2+^ levels and lipid peroxydation accumulation in HT1080 cells (**Figures 4F–I**). Western blotting demonstrated that TRIM22 inhibition significantly decreased heme oxygenase (HMOX1) protein levels, while increasing the protein levels of ferroptosis suppressor protein 1 (FSP1, also known as AIMF2), both of which are regulatory genes of ferroptosis (**Figure 4J**). Besides TRIM proteins, we individually knocked down PML, PARP9, PTGS2, CHAC1, and ATF3 in HT1080 through siRNA silencing. We observed that depletion of these proteins did not affect or enhance intracellular Fe^2+^ levels and lipid peroxydation accumulation in IFNβ-treated HT1080 cells. These results indicate that TRIM22 plays an essential role in IFNβ-mediated ferroptosis.

### 3.4 IFNβ enhances RSL3-induced ferroptosis in HT1080 cells

**Figure 5:**
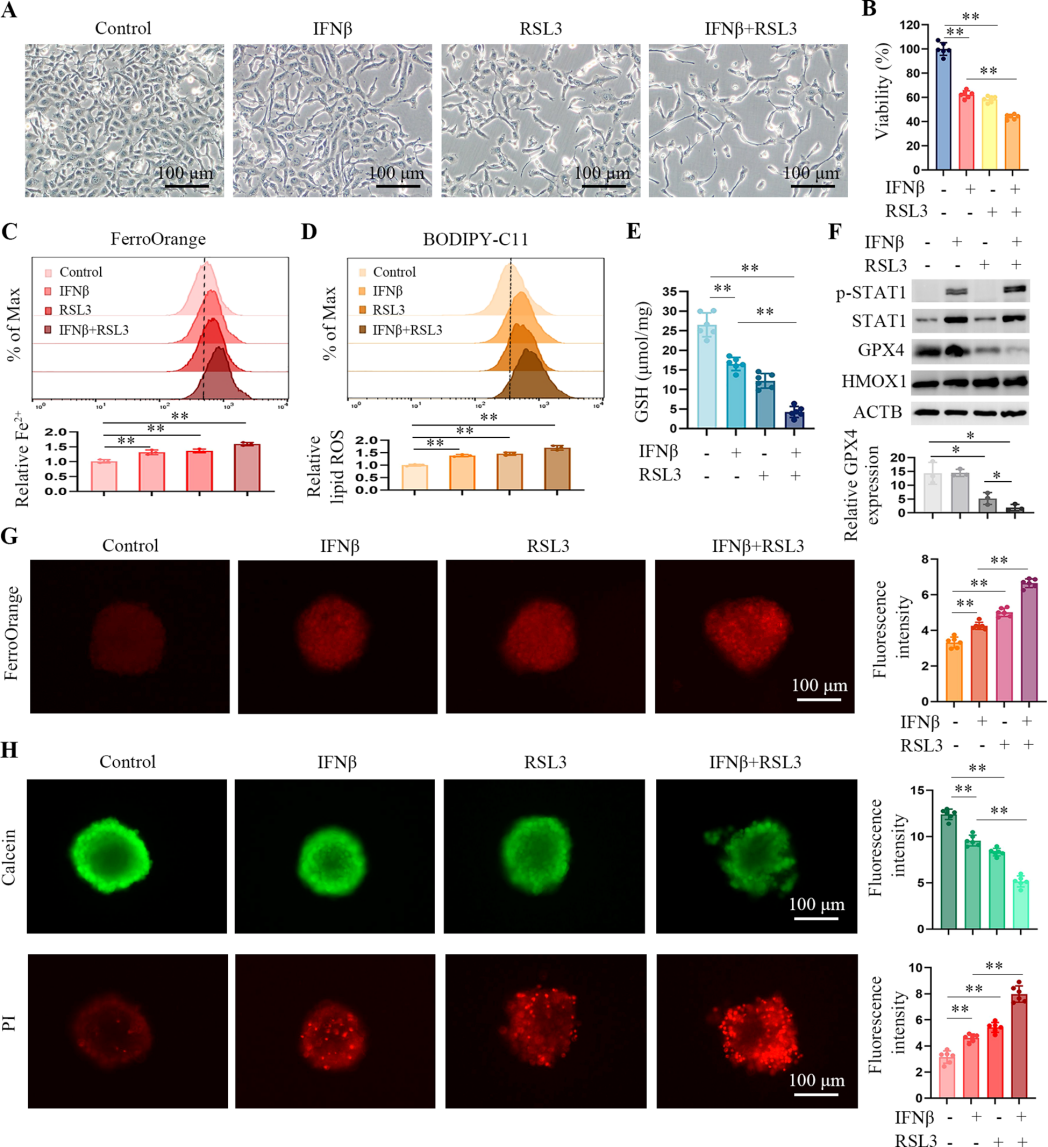
IFNβ treatment enhances RSL3-induced ferroptosis in HT1080 cells. **(A)** Representative images by inverted light microscopy showing morphology changes in HT1080 cells treated with IFNβ (20 ng/mL), RSL3 (0.5 μM), or their combination for 48 h. Scale bar, 100 μm. One representative experiment of three independent experiments is shown. **(B)** CCK-8 assay showing the response of HT1080 cells to IFNβ or RSL3 or their combination for 48 h. Data indicated as mean ± S.D. (n = 6 replicates). One representative experiment of three independent experiments is shown. **(C, D)** Flow cytometric analysis of intracellular Fe^2+^ and lipid ROS levels in HCT1080 cells treated with IFNβ, RSL3, or their combination for 24 h. Data indicated as mean ± S.D. (n = 3 experiments). **(E)** Levels of GSH in HCT1080 cells treated with IFNβ, RSL3, or their combination for 24 h. Data indicated as mean ± S.D. (n = 6 experiments). **(F)** Representative images of western blotting analysis showing the protein levels of STAT1, GPX4 and HMOX1 in HCT1080 cells treated with IFNβ, RSL3, or their combination for 24 h. **(G)** Representative images by immunofluorescence showing intracellular Fe^2+^ levels in HT1080 spheroids treated with IFNβ, RSL3, or their combination for 6 h. Bar plots represent the fluorescence intensity of five spheres in one experiment. Data indicated as mean ± S.D. (n = 6 experiments). **(H)** Representative images of HT1080 spheroids stained with Calcein-AM (green) and PI (red). Data indicated as mean ± S.D. (n = 6 experiments). Statistical significance relative to mock conditions is indicated as *P < 0.05, **P < 0.01.

Our data shows that IFNβ only induces ferroptosis up to a certain amount. We wondered if IFNβ could augment the cytotoxicity of the ferroptosis inducer. HT1080 cells were treated with IFNβ, RSL3, or IFNβ combination with RSL3. Compared with IFNβ alone, the combination treatment enhanced the death of HT1080 cells (**Figures 5A, B**). Furthermore, the combination treatment increased intracellular Fe^2+^ (**Figure 5C**) and lipid peroxidation (**Figure 5D**). The GSH levels were significantly lower in the two compounds’ co-treated cells (**Figure 5E**). GPX4 is targeted by the ferroptosis inducer RSL3 (2). **Figure 5F** demonstrates that while IFNβ treatment alone did not significantly affect GPX4 levels, simultaneous exposure to IFNβ and RSL3 further inhibited GPX4 levels compared to RSL3 treatment alone. This suggests that IFNβ may enhance RSL3-induced ferroptosis, possibly due to the increased inhibition of GPX4. Moreover, we observed similar results in 786 cells, IFNβ enhanced the ferroptosis inducing effect of RSL3 (**Supplementary Figure S6**).

*In vitro*, 3D cancer cell culture exhibits mimic profiles to solid tumors. 3D spheroids are more appropriate for cancer drug screening (19). Therefore, we used ultra-low attachment round-bottom 96-well plates to develop HT1080 3D spheroids. Intracellular iron levels were determined in HT1080 spheroids by using a FerroOrange probe. The spheroids co-treated with IFNβ and RSL3 had stronger fluorescence than those treated with IFNβ or RSL3 alone (**Figure 5G**). To further confirm the proportion of live/dead cells in the cell spheres, we stained spheroids with calcein-AM (green) and PI (red). As shown in **Figure 5H**, compared to the IFNβ or RSL3-treated spheroids, co-treated spheroids caused higher levels of PI (dead cells) and lower calcein-AM (live cells) signal. These findings suggest that following treatment with IFNβ and RSL3 increased ferroptosis in HT1080 cell lines.

### 3.5 Combination treatment with IFNβ and RSL3 promotes ferroptosis in the HT1080 xenograft nude mouse model

**Figure 6:**
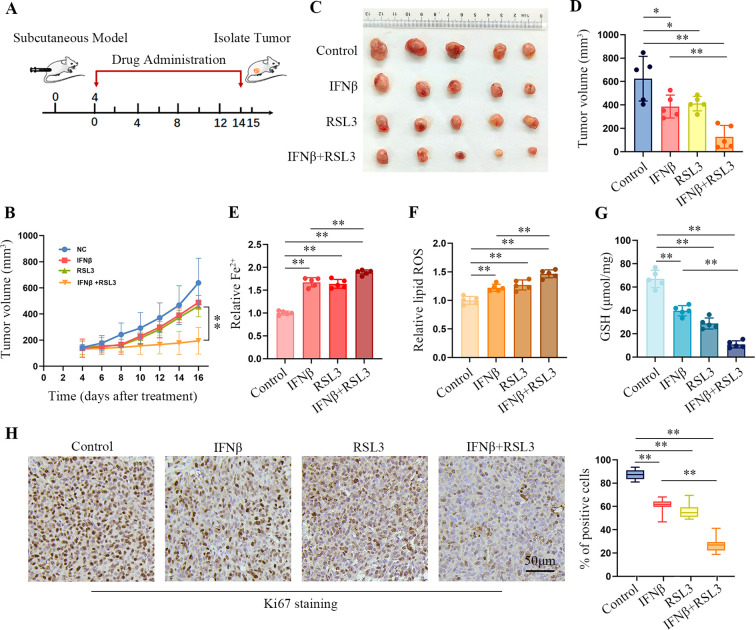
IFNβ enhances the toxicity of RSL3 in HT1080-derived xenograft tumors. **(A)** Schematic of the anti-tumor effect of the combination of IFNβ (100 ng per mouse) and RSL3 (2.5 mg/kg) in an HT1080 subcutaneous tumor model. **(B)** Growth curves of the xenograft tumors in BALB/c nude mice (n = 5 mice per group). **(C)** Representative images of HT1080 xenograft tumors in BALB/c nude mice injected with IFNβ, RSL3, or their combination. **(D)** Tumor volume of HT1080 xenograft tumors in BALB/c nude mice injected with IFNβ, RSL3, or their combination. n = 5 mice per group, mean ± SD. **(E–G)** Levels of intracellular Fe^2+^, lipid ROS, and GSH in HT1080 xenograft tumors treated with IFNβ, RSL3, or their combination. n = 5 mice per group, mean ± SD. **(H)** Representative image of Ki67 immunohistochemical staining in the HT1080 xenograft tumor tissues treated with IFNβ, RSL3, or their combination. Scale bars: 50 μm. n = 5 mice per group. Statistical significance relative to mock conditions is indicated as *P < 0.05, **P < 0.01.

We established an HT1080 xenograft nude mouse model to explore whether IFNβ and RSL3 combination treatment *in vivo* enhanced ferroptosis. **Figure 6A** shows the schematic of tumor inoculation and systemic injection. All the mice survived after cell implantation and drug injection. After 16 days, the tumor size gradually increased in the control group. However, administration of IFNβ, RSL3, or IFNβ in combination with RSL3 led to a decrease in tumor size (**Figure 6B**). Moreover, combination treatment improved the efficacy of RSL3 in suppressing tumor growth (**Figures 6C, D**). In addition, we found that co-treatment significantly enhanced the intracellular Fe^2+^ and lipid peroxydation levels (**Figures 6E, F**) but decreased the amount of GSH compared to IFNβ or RSL3 administration (**Figure 6G**). Immunohistochemical staining assays also show lower levels of Ki67 in the co-treatment group (**Figure 6H**). These results further support that combination treatment with IFNβ and RSL3 increased HT1080 ferroptosis.

## 4 Discussion

The cGAS–STING pathway implicates host defense, inflammation, and tumor immunity (4, 7, 20). Previous studies have reported that this pathway also links to cell death processes, including apoptosis, pyroptosis, and necroptosis (21). Our study found that the cGAS–STING pathway produced IFNβ sensitized tumors to ferroptosis. IFNβ induced intracellular Fe^2+^ and lipid peroxydation levels while decreasing GSH levels in cancer cells.

Cytokines, such as interferons (IFNs), interleukin 6 (IL-6), and platelet-derived growth factor (PDGF), could activate the STAT1/STAT3 pathway (22). IFNβ binds to a heterodimeric receptor consisting of IFNα receptor 1 (IFNAR1) and IFNAR2, and then engages the JAK kinases to phosphorylate STAT1/STAT3 (23). Our study used the activation of the STAT1/STAT3 pathway to indicate IFNβ exposure. STAT1 has been reported to be a ferroptosis inducer protein, which induces the expression of cyclooxygenase-2 (COX-2) (14). In contrast, STAT1 inhibition observed here did not affect IFNβ-mediated intracellular Fe^2+^ and lipid peroxydation level increment. Pharmacological or genetic inhibition of STAT3 could block erastin-induced ferroptosis in pancreatic ductal adenocarcinoma (PDAC) cells (24). However, Ouyang et al. reported that knockdown of STAT3 promotes ferroptosis in gastric cancer cells (13). Our data show that STAT3 inhibition could enhance IFNβ-mediated ferroptosis in HT1080 cells. These findings are in line with Ouyang colleagues, but the modest difference may also suggest that the impact of STAT3 on ferroptosis isn’t as clear, and more data is still needed to confirm.

Most TRIM family proteins have E3 ubiquitin ligase activities (25). IFNs have been reported to stimulate several TRIM genes, such as PML, TRIM8, TRIM21, and TRIM22 (26). Our data showed that IFNβ treatment significantly stimulated TRIM21 expression. TRIM21 depletion has been reported to suppress doxorubicin-induced ferroptosis through mediating activation of the p62-Keap1-NRF2 antioxidant pathway (27). We silenced the expression of TRIM21 and found that TRIM21 silencing did not affect IFNβ-mediated intracellular Fe^2+^ and lipid peroxydation level increment.

Like TRIM21, TRIM22 is induced by IFNβ. We demonstrated that IFNβ strongly induced the expression of TRIM22. Knockdown TRIM22 reduced IFNβ-induced intracellular Fe^2+^ levels, lipids oxidation rates, and HMOX1 protein levels. However, it led to an increase in FSP1 protein levels. HMOX1 plays a role in iron and ROS homeostasis. Excessive activation of HMOX1 led to heme degradation and the release of free iron (28, 29). FSP1 is an inhibitor of ferroptosis (30, 31). Ubiquitination modification is one of the regulatory mechanisms of FSP1 (32). The ubiquitin ligase TRIM22 could possibly be involved in maintaining the stability of the FSP1 protein. Although further research is needed to clarify how TRIM22 was upregulated by IFNβ and the role of TRIM22 in HMOX1 and FSP1 regulation, we hypothesized that TRIM22 served as a regulator of IFNβ-mediated ferroptosis.

RSL3 is a known ferroptosis inducer that reduces GPX4 activity and inhibits the clearance capacity of lipid peroxides (33). Our results demonstrate that the combination treatment of IFNβ and RSL3 leads to increased intracellular Fe^2+^ levels and lipid peroxidation in HT1080 cells, both *in vitro* and *in vivo*. In addition, IFNβ can enhance the effect of RSL3 in reducing GPX4 levels. These findings suggest that IFNβ enhances the cytotoxic effect of ferroptosis-inducing compounds, providing a strong rationale for considering IFNβ in cancer therapy. Future research should focus on monitoring and evaluating how IFNβ enhances the cytotoxic effect of other ferroptosis inducers. Furthermore, beyond ferroptosis, IFNβ triggers apoptosis as a cell death mechanism, as confirmed by previous studies (9). While our investigation specifically examined the ferroptotic pathway induced by IFNβ, we acknowledge the scope for exploring additional cell death mechanisms. Future therapeutic strategies may benefit from combining IFNβ with both ferroptosis inducers and apoptosis-promoting agents to enhance tumor treatment efficacy.

## 5 Conclusion

In summary, our study reveals that IFNβ promotes ferroptosis in cancer cells. IFNβ disturbs the expression of several ferroptosis-related genes. TRIM22 inhibition moderates IFNβ-mediated ferroptosis. In addition, we report that IFNβ enhances RSL3-induced ferroptosis. These findings expand the knowledge on IFNβ role in cancers.

## Data Availability

The data presented in this study are deposited in the GEO repository under accession number GSE288379.
