# Supplementary material for: IFNβ drives ferroptosis through elevating TRIM22 and promotes the cytotoxicity of RSL3
Source: Front Immunol. 2025 Feb 5;16:1535554. doi: 10.3389/fimmu.2025.1535554 (PMC11836015; doi:10.3389/fimmu.2025.1535554)
Supplement: Supplementary file 1 [file DataSheet1.pdf]

FIGURE S1 Representative images of the fluorescent probe C11-BODIPY 581/591. HT1080 cells were treated for 24 h with or without IFNβ (20 ng/mL). The scale bar represents 25 μm.

FIGURE S2 IFNβ treatment induces ferroptosis in tumor cells. **(A)** Representative images by inverted light microscopy show morphology changes in 786-O and HCT116 cells treated with IFNβ (20 ng/mL) for 48 h. Scale bar: 200 μm**. (B)** CCK-8 assay showing the response of 786-O and HCT116 cell lines to IFNβ (20 ng/mL). **(C, D)** Flow cytometric analysis of intracellular Fe^{2+} and lipid ROS levels in 786-O and HCT116 cells treated with or without 20 ng/mL IFNβ for 24 h. n = 6, mean ± SD, statistical significance relative to mock conditions are indicated as * $P < 0.05$ and ** $P < 0.01$.

FIGURE S3 Representative pictures acquired by transmission electron microscopy. HT1080 or 4T1 cells were treated for 24 h with or without IFNβ (20 ng/mL). The scale bar represents 1 μm.

FIGURE S4 IFNβ treatment changes ferroptosis-related gene expression in 4T1 cells. **(A)** Bar graph of functional enrichment analysis by Metascape. **(B)** Venn diagrams showing the differentially expressed genes (DEGs) that overlapped among ferroptosis regulators. **(C)** Heatmap of ferroptosis regulators detected by the RNA-seq. The heatmap shows a fold-change of ferroptosis genes that range from zero to 100 between IFNβ untreated and treated 4T1 cells (n = 3 samples).

FIGURE S5 Activation of the cGAS-STING signaling pathway promotes ferroptosis in heart tissue. **(A)** ELISA analysis showing the IFNβ levels in the serum of cGAMP untreated or treated mice (n = 6). **(B)** RT-qPCR analysis of the mRNA level of IFNB1 in heart tissues of cGAMP untreated or treated mice (n = 6). **(C)** Western blotting analysis showing the total protein levels and phosphorylation status of STAT1 and STAT3 in mice primed with or without cGAMP (n = 3). **(D, E, F)** Levels of intracellular iron, lipid ROS, and GSH in heart tissues of cGAMP untreated or treated mice. **(G)** Relative mRNA levels of ferroptosis regulators expression in heart tissues of cGAMP untreated or

treated mice. n = 6 per group, mean ± SD. Statistical significance relative to mock conditions is indicated as * $P < 0.05$ and ** $P < 0.01$.

FIGURE S6 IFNβ treatment enhances RSL3-induced ferroptosis in 786-O cells. **(A)** Representative images by inverted light microscopy showing morphology changes in 786-O cells treated with IFNβ (20 ng/mL), RSL3 (0.5 μM), or their combination for 48 h. Scale bar, 100 μm. One representative experiment of three independent experiments is shown. **(B)** CCK-8 assay showing the response of 786-O cells to IFNβ or RSL3 or their combination for 48 h. Data indicated as mean ± S.D. (n = 6 replicates). One representative experiment of three independent experiments is shown. **(C)** Flow cytometric analysis of intracellular lipid ROS levels in 786-O cells treated with IFNβ, RSL3, or their combination for 24 h. Data indicated as mean ± S.D. (n = 3 experiments). **(D)** Representative images of western blotting analysis showing the protein levels of STAT1, GPX4 and HMOX1 in 786-O cells treated with IFNβ, RSL3, or their combination for 24 h.

Figure S1

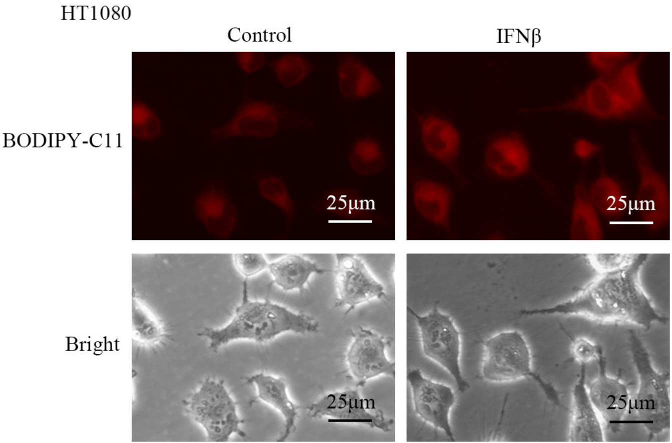

Figure S2

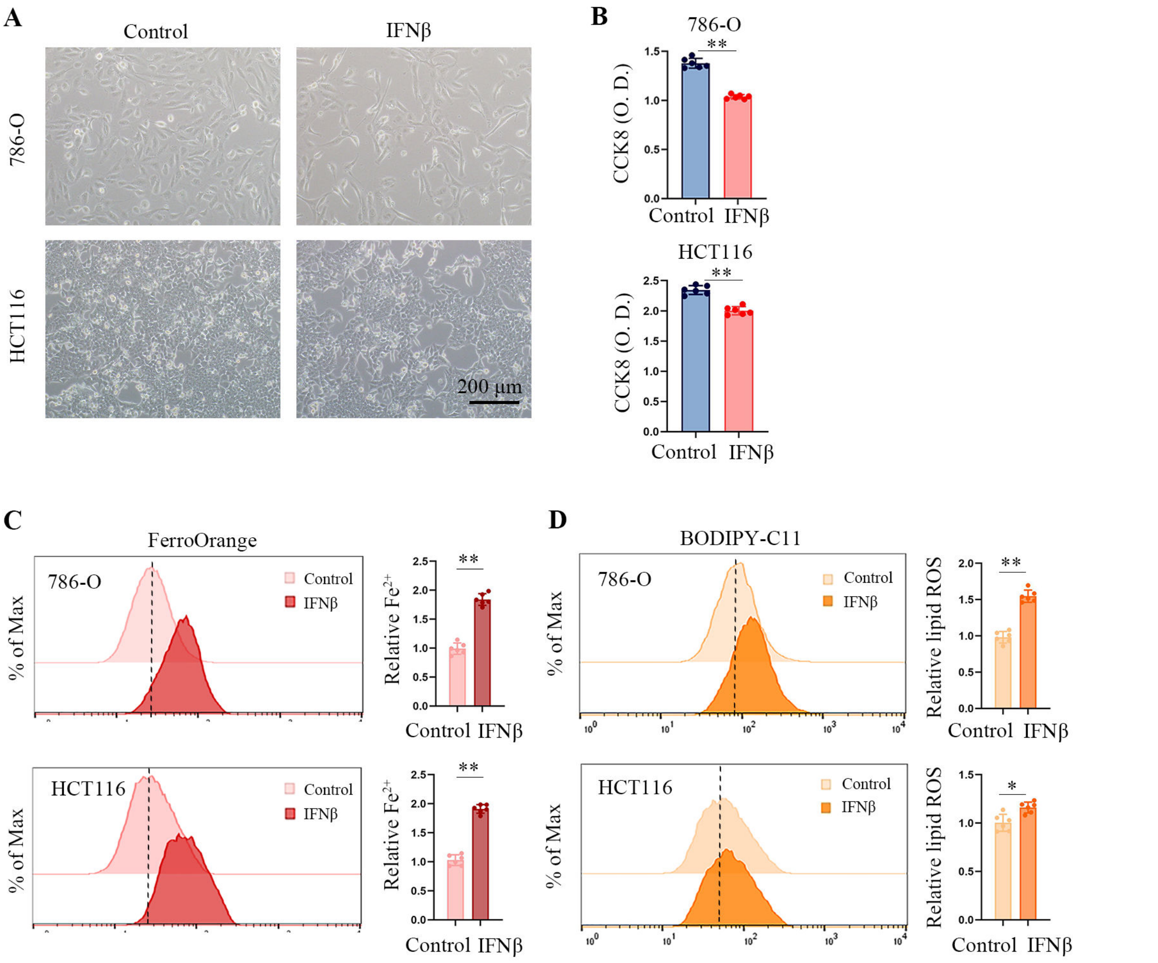
A
Control
IFNβ
786-O
HCT116
200 μm
B
786-O
CCK8 (O. D.)
1.5
1.0
0.5
0.0
Control
IFNβ
**
HCT116
CCK8 (O. D.)
2.5
2.0
1.5
1.0
0.5
0.0
Control
IFNβ
**
C
FerroOrange
786-O
% of Max
Control
IFNβ
Relative Fe2+
2.5
2.0
1.5
1.0
0.5
0.0
**
Control
IFNβ
HCT116
% of Max
Control
IFNβ
Relative Fe2+
**
Control
IFNβ
D
BODIPY-C11
786-O
% of Max
Control
IFNβ
Relative lipid ROS
2.0
1.5
1.0
0.5
0.0
**
Control
IFNβ
HCT116
% of Max
Control
IFNβ
Relative lipid ROS
1.5
1.0
0.5
0.0
*
Control
IFNβ

Figure S3

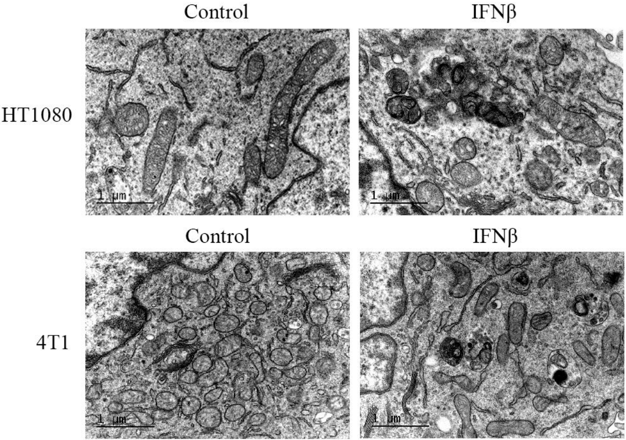

Figure S4

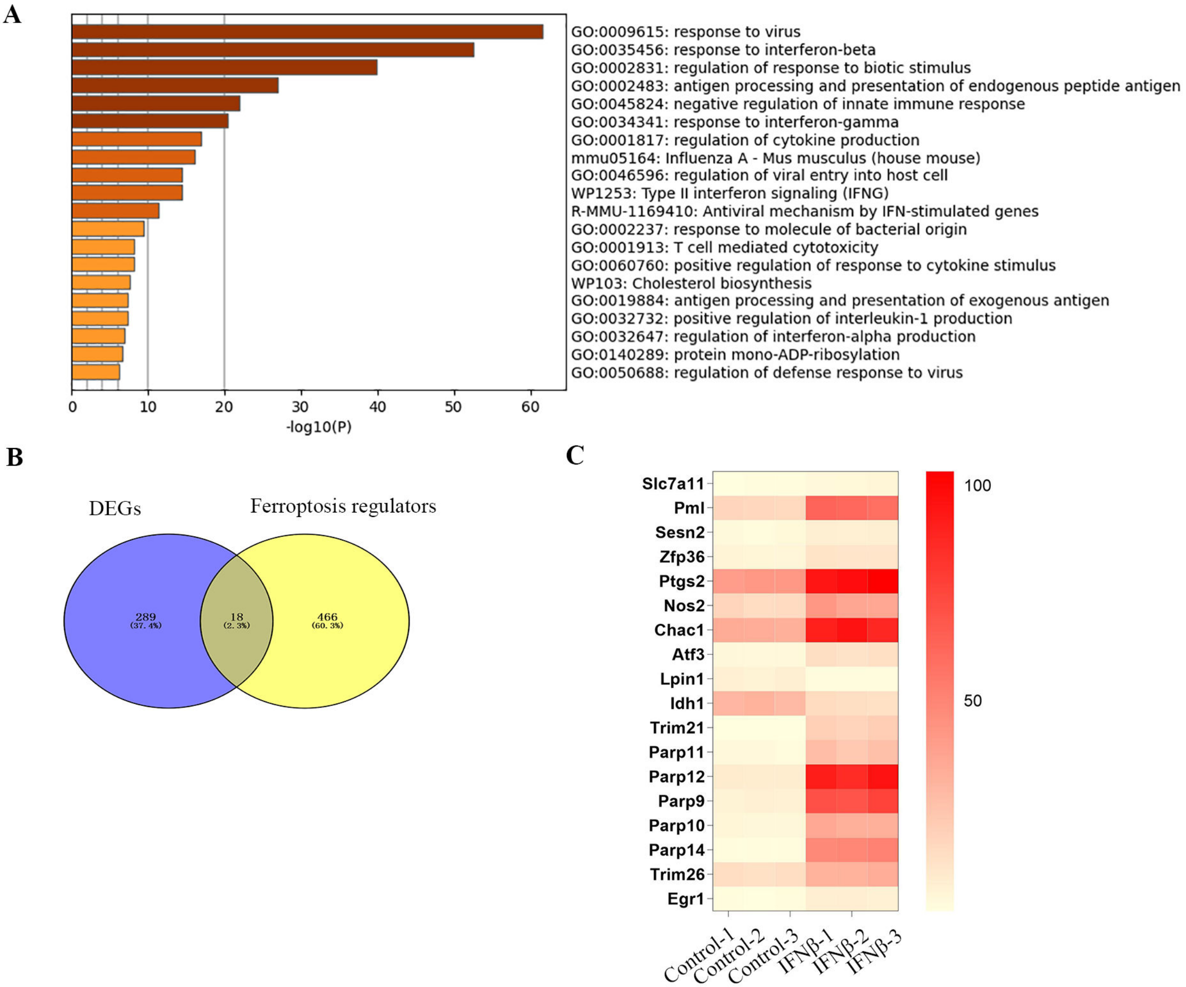
A
GO:0009615: response to virus
GO:0035456: response to interferon-beta
GO:0002831: regulation of response to biotic stimulus
GO:0002483: antigen processing and presentation of endogenous peptide antigen
GO:0045824: negative regulation of innate immune response
GO:0034341: response to interferon-gamma
GO:0001817: regulation of cytokine production
mmu05164: Influenza A - Mus musculus (house mouse)
GO:0046596: regulation of viral entry into host cell
WP1253: Type II interferon signaling (IFNG)
R-MMU-1169410: Antiviral mechanism by IFN-stimulated genes
GO:0002237: response to molecule of bacterial origin
GO:0001913: T cell mediated cytotoxicity
GO:0060760: positive regulation of response to cytokine stimulus
WP103: Cholesterol biosynthesis
GO:0019884: antigen processing and presentation of exogenous antigen
GO:0032732: positive regulation of interleukin-1 production
GO:0032647: regulation of interferon-alpha production
GO:0140289: protein mono-ADP-ribosylation
GO:0050688: regulation of defense response to virus
0
10
20
30
40
50
60
-log10(P)
B
DEGs
Ferroptosis regulators
289
(37.4%)
18
(2.3%)
466
(60.3%)
C
Slc7a11
Pml
Sesn2
Zfp36
Ptgs2
Nos2
Chac1
Atf3
Lpin1
Idh1
Trim21
Parp11
Parp12
Parp9
Parp10
Parp14
Trim26
Egr1
100
50
Control-1
Control-2
Control-3
IFNβ-1
IFNβ-2
IFNβ-3

Figure S5

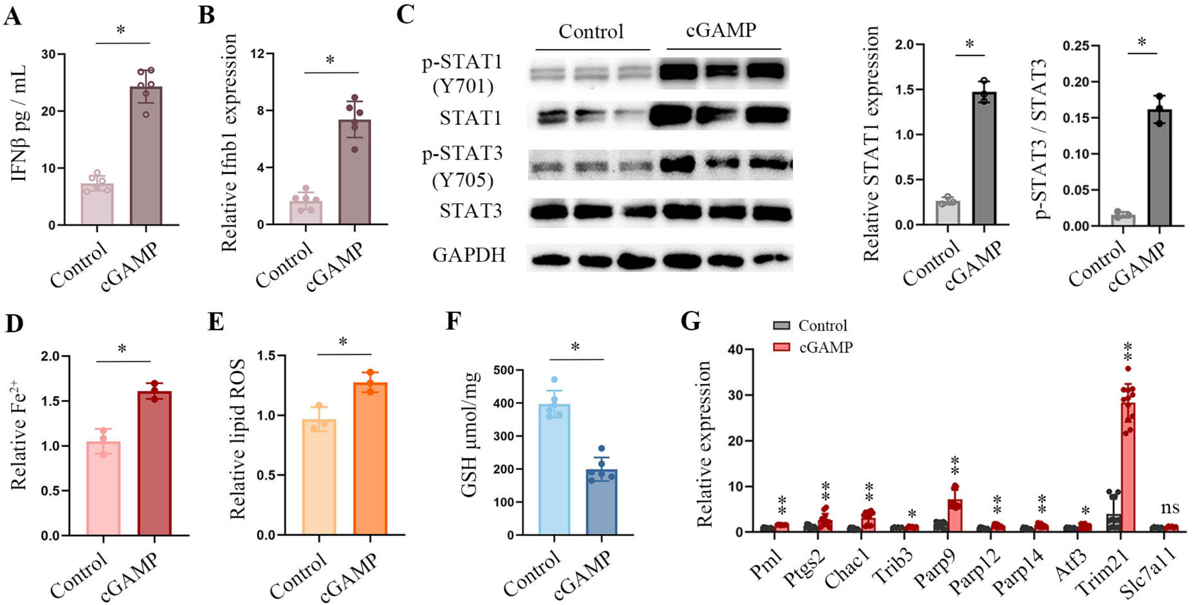

A
IFNβ pg / mL
30
20
10
0
*
Control
cGAMP
B
Relative Ifnb1 expression
12
8
4
0
*
Control
cGAMP
C
Control
cGAMP
p-STAT1
(Y701)
STAT1
p-STAT3
(Y705)
STAT3
GAPDH
Relative STAT1 expression
2.0
1.5
1.0
0.5
0.0
*
Control
cGAMP
p-STAT3 / STAT3
0.25
0.20
0.15
0.10
0.05
0.00
*
Control
cGAMP
D
Relative Fe2+
2.0
1.5
1.0
0.5
0.0
*
Control
cGAMP
E
Relative lipid ROS
1.5
1.0
0.5
0.0
*
Control
cGAMP
F
GSH μmol/mg
500
400
300
200
100
0
*
Control
cGAMP
G
Control
cGAMP
Relative expression
40
30
20
10
0
**
**
**
*
**
**
**
*
**
ns
Pml
Ptgs2
Chac1
Trib3
Parp9
Parp12
Parp14
Atf3
Trim21
Slc7a11

Figure S6

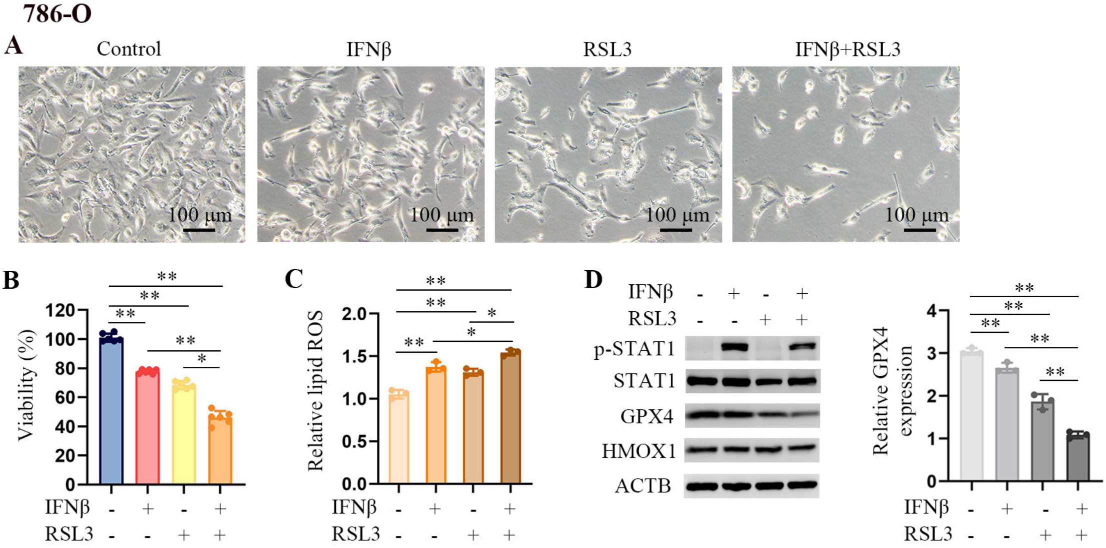
786-O
A
Control
IFNβ
RSL3
IFNβ+RSL3
100 μm
100 μm
100 μm
100 μm
B
Viability (%)
120
100
80
60
40
20
0
IFNβ - + - +
RSL3 - - + +
C
Relative lipid ROS
2.0
1.5
1.0
0.5
0.0
IFNβ - + - +
RSL3 - - + +
D
IFNβ - + - +
RSL3 - - + +
p-STAT1
STAT1
GPX4
HMOX1
ACTB
Relative GPX4 expression
4
3
2
1
0
IFNβ - + - +
RSL3 - - + +
