# Supplementary material for: IFNβ drives ferroptosis through elevating TRIM22 and promotes the cytotoxicity of RSL3
Source: Front Immunol. 2025 Feb 5;16:1535554. doi: 10.3389/fimmu.2025.1535554 (PMC11836015; doi:10.3389/fimmu.2025.1535554)
Supplement: Supplementary file 2 [file DataSheet2.pdf]

Supplementary Table S1: siRNA sequences used for gene knockdown

siRNA	Forward Primer(5'-3')	Reverse Primer(5'-3')
si-STAT1#1	CUGGAAGAUUUACAAGAUGAATT	UUCAUCUUGUAAAUCUUCCAGTT
si-STAT1#2	CCCUGAAGUAUCUGUAUCCAATT	UUGGAUACAGAUACUUCAGGGTT
si-STAT3#1	GCCUCUCUGCAGAAUUCAATT	UUGAAUUCUGCAGAGAGGCTG
si-STAT3#2	GCGUCCAGUUCACUACUAATT	UUAGUAGUGAACUGGACGCCG
si-TRIM21#1	GCUCCCUCAUCUACUCCUUTT	AAGGAGUAGAUGAGGGAGCCA
si-TRIM21#2	GCAGGAGUUGGCUGAGAAGTT	CUUCUCAGCCAACUCCUGCTT
si-TRIM22#1	CCACUUCGAAUGUUGCUAUTT	AUAGCAACAUUCGAAGUGGCA
si-TRIM22#2	GCUACUGGGUUAUAGGAUUTT	AAUCCUAUAACCCAGUAGCCA
si-Control	UUCUCCGAACGUGUCACGUDTDT	ACGUGACACGUUCGGAGAADTDT

Supplementary Table S2: RT-PCR primers used in this study

Gene	Forward Primer(5'-3')	Reverse Primer(5'-3')
m*Stat1*	TGCCGAGAACATACCAGAGA	CCGGGACATCTCATCAAACT
m*Stat3*	GTACCACCAAGGCCTCAAGA	TGGCGGCTTAGTGAAGAAGT
m*Ifnb1*	CCCTATGGAGATGACGGAGA	CTGTCTGCTGGTGGAGTTCA
m*PML*	TGGACGATAGCAGCAGTGAG	ATGGTGGTGAGGAAGCTGAG
m*PTGS2*	CGGAGAGAGTTCATCCCTGA	ACCTCTCCACCAATGACCTG
m*CHAC1*	GCAGGGAGACACCTTCCATA	GTACTTCAGGGCCTCGTTCA
m*PARP9*	AGCAGGTCCCACATCAGTTC	ACATCTAAGGCCCCAGGACT
m*PARP12*	GGGGGAAGGAAGTAGACGAG	GGCATCTCTCGCAAAGTAGC
m*PARP14*	CTTCCATGGGACAGAGGCTA	CTCCCATTGGTGTCTGGTCT
m*ATF3*	ACAACAGACCCCTGGAGATG	CCTTCAGCTCAGCATTCACA
m*TRIM21*	GAGGATTCGTGGTTCAGAGC	ACGTTCTGATGGGTGTCTCC
m*Actb*	GTCCCTCACCCTCCCAAAAG	GCTGCCTCAACACCTCAACCC
m*Gapdh*	GGTGAAGGTCGGTGTGAACG	CTCGCTCCTGGAAGATGGTG
h*STAT1*	CAAAGTCATGGCTGCTGAGA	GCAGGTTGTCTGTGGTCTGA
h*STAT3*	CCTGGTGTCTCCACTGGTCT	GGTTCAGCACCTTCACCATT
h*STING1*	TTGGCTGAGTGTGTGGAGTC	AGGCACTCAGCAGAACCAAG
h*IFNB1*	ACTGCCTCAAGGACAGGATG	AGCCAGGAGGTTCTCAACAA
h*PML*	AGCAGCAGTGAGTCCAGTGA	ACACGGCCTTGGAGTAGATG
h*PTGS2*	ATTCTTTGCCCAGCACTTCA	CAGCAAACCGTAGATGCTCA
h*CHAC1*	TGGTGACGCTCCTTGAAGAT	AATGCCTTCAGTGGTTGGTC
h*PARP9*	TACAGGAGGAAATGGCAAGG	GAGTTGGAGGCACAGGACAT
h*PARP12*	ACCAGTGGCAAAAAGGACAG	CATCTCGGGCAAAGTAGCTC
h*PARP14*	GAGCTGCTGCCTAGTGATCC	TCATTGTCTGGCCATTCTTG
h*ATF3*	GTGCCGAAACAAGAAGAAGG	TGGAGTCCTCCCATTCTGAG
h*TRIM21*	AAAGCAGGAGTTGGCTGAGA	CTCTTTCTCCCCCAGGATTC
h*ACTB*	TGACGTGGACATCCGCAAAG	CTGGAAGGTGGACAGCGAGG
h*GAPDH*	AGAAGGCTGGGGCTCATTTG	AGGGGCCATCCACAGTCTTC

Supplementary Table S3. Antibodies information used in this study

Antibodies	Souce	Identifier	Dilution
Phospho-STAT1 (Tyr701)	Cell Signaling Technology	9167S	1:1000
STAT1	Abclonal	A19563	1:1000
Phospho-STAT3 (Tyr705)	Cell Signaling Technology	9145S	1:1000
STAT3	Cell Signaling Technology	4904S	1:1000
COX2	Cell Signaling Technology	12282P	1:1000
TREX1	Santa Cruz	sc-133112	1:500
SLC7A11	Abclonal	A2413	1:1000
PML	Proteintech	21041-1-AP	1:1000
PARP9	Proteintech	17535-1-AP	1:1000
CHAC1	Proteintech	15207-1-AP	1:1000
TRIM21	Proteintech	12108-1-AP	1:1000
TRIM22	Proteintech	13744-1-AP	1:1000
AIFM2/FSP1	Proteintech	20886-1-AP	1:1000
HMOX1	Proteintech	10701-1-AP	1:1000
GPX4	Abcam	ab125066	1:2000
GAPDH	Cell Signaling Technology	2118S	1:1000
ACTB	Sigma-Aldrich	A2228	1:4000

Supplementary Table S4. Twenty-five ferroptosis-related genes and its fold-differences of HT1080 cells

Gene	NC	IFNβ	Fold change	p-value
IDO1	0.04±0.03	1.43±0.07	34.29	1.22E-04
DPP4	0.00±0.01	0.02±0.01	27.75	1.57E-02
PARP9	3.31±0.07	67.07±0.66	20.24	2.20E-05
PARP14	5.27±0.11	89.06±2.01	16.90	1.38E-04
PARP10	3.94±0.06	49.09±1.07	12.46	1.35E-04
PARP12	2.18±0.17	17.90±0.52	8.21	8.70E-05
PARP15	0.01±0.01	0.06±0.02	7.27	3.06E-02
TRIM21	14.86±0.38	76.80±0.27	5.17	1.22E-08
PML	7.46±0.31	33.30±0.39	4.46	1.99E-07
IL6	0.27±0.09	0.94±0.12	3.45	2.33E-03
CA9	0.24±0.02	0.80±0.30	3.35	6.01E-02
LGMN	4.78±0.18	15.75±0.29	3.29	4.49E-06
SLC25A28	4.21±0.15	13.78±0.15	3.27	1.86E-07
RICTOR	3.18±0.11	8.13±0.31	2.56	3.14E-04
GCH1	4.14±0.19	10.31±0.37	2.49	1.18E-04
STING1	9.71±0.41	23.62±0.65	2.43	2.97E-05
ATF3	1.15±0.07	32.36±1.34	2.38	2.58E-04
SOCS1	1.81±0.23	4.11±0.21	2.28	2.48E-04
TRIM26	12.19±0.46	26.00±0.27	2.13	1.23E-05
CHMP5	32.59±1.16	68.91±0.61	2.11	1.67E-05
ASMTL-AS1	1.06±0.14	2.24±0.25	2.12	3.95E-03
MAPKAP1	26.90±0.36	12.89±0.38	0.48	1.50E-06
ACO1	20.09±0.56	8.98±0.49	0.45	1.65E-05
FABP4	1.52±0.27	0.65±0.18	0.43	1.29E-02
NT5DC2	24.22±0.75	8.63±0.11	0.36	4.78E-04

Supplementary Table S5. Eighteen ferroptosis-related genes and its fold-differences of 4T1 cells

Gene	NC	IFNβ	Fold change	p-value
Slc7a11	1.65±0.30	3.80±0.43	2.30	3.26E-03
Pml	16.41±0.64	60.8±2.28	3.70	4.00E-04
Sesn2	3.09±0.46	7.1±0.19	2.29	1.42E-03
Zfp36	4.83±0.32	11.19±0.52	2.32	2.13E-04
Ptgs2	41.21±1.00	98.28±4.60	2.38	1.44E-03
Nos2	15.73±1.66	38.44±3.61	2.44	2.85E-03
Chac1	33.48±0.81	91.04±3.92	2.72	1.04E-03
Atf3	3.85±0.23	12.89±0.78	3.35	1.23E-03
Lpin1	6.72±0.84	2.35±0.28	0.35	6.78E-03
Idh1	30.07±1.65	13.94±1.16	0.46	3.07E-04
Parp11	3.20±0.53	25.18±2.55	7.88	3.32E-03
Parp12	8.28±0.22	90.99±4.82	10.98	1.10E-03
Parp9	6.31±0.50	72.16±3.81	11.44	9.40E-04
Parp10	4.28±0.13	33.53±1.69	7.83	1.05E-03
Parp14	1.72±0.10	49.35±1.48	28.76	3.01E-04
Trim21	1.26±0.16	19.49±1.13	15.46	1.05E-03
Trim26	13.62±0.66	32.36±1.34	2.38	2.58E-04
Egr1	1.69±0.24	7.16±0.66	4.25	2.05E-03
