# Supplementary material for: IFNβ drives ferroptosis through elevating TRIM22 and promotes the cytotoxicity of RSL3
Source: Front Immunol. 2025 Feb 5;16:1535554. doi: 10.3389/fimmu.2025.1535554 (PMC11836015; doi:10.3389/fimmu.2025.1535554)

Full and uncropped western blot for Figure 2F

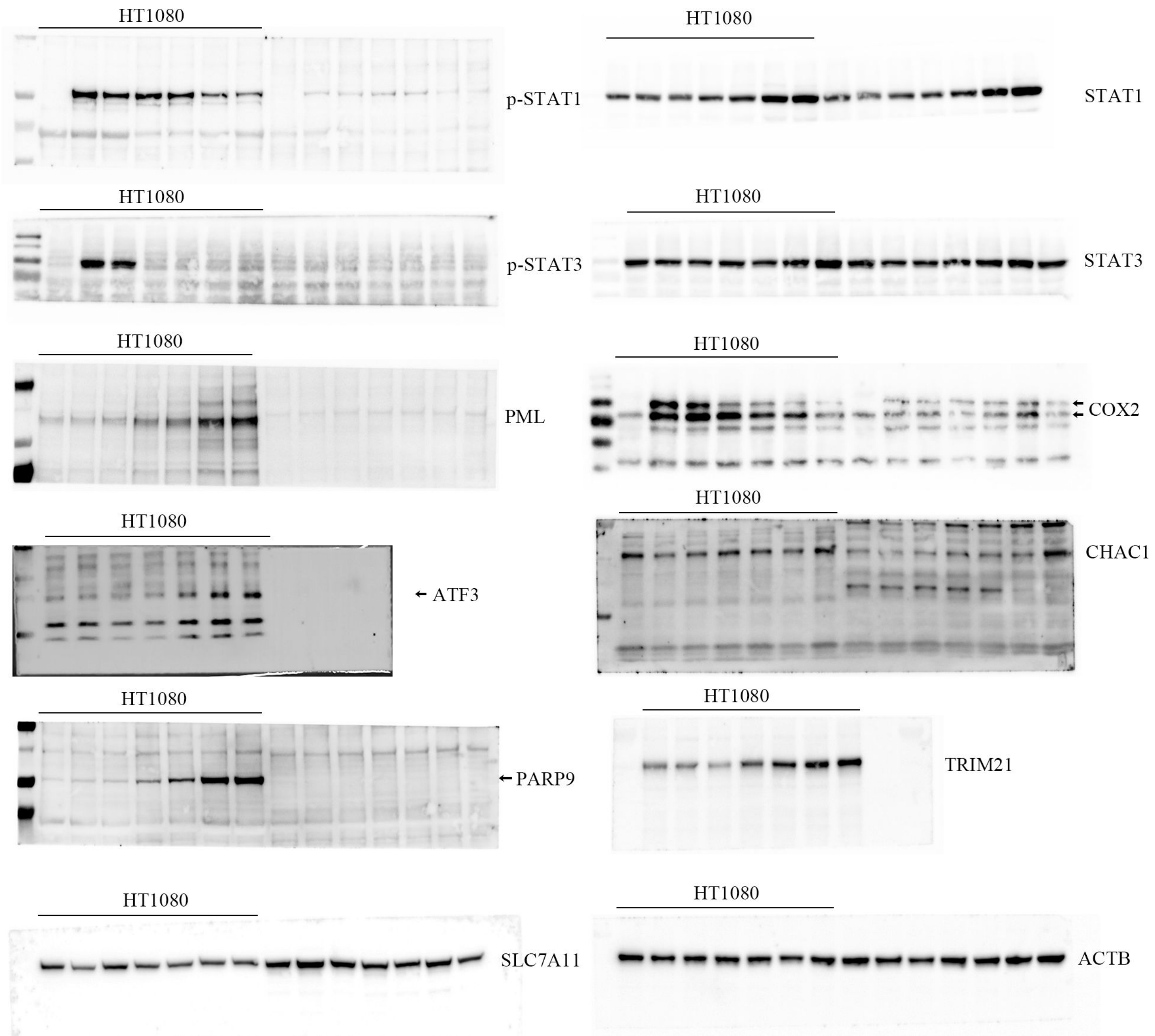

Full and uncropped western blot for Figure 2G

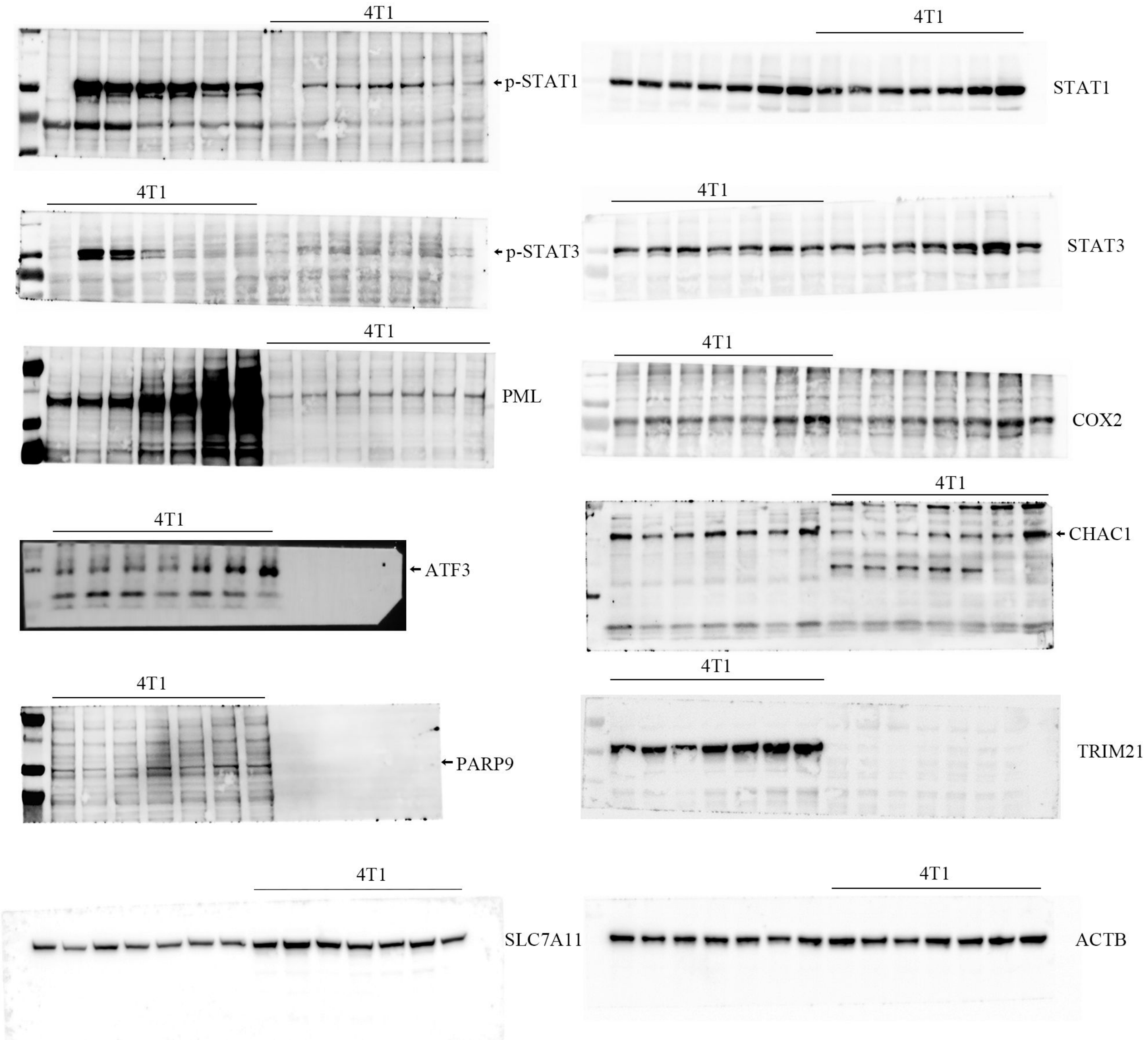

Full and uncropped western blot for Figure 3A

STAT1

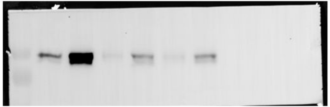

ACTB

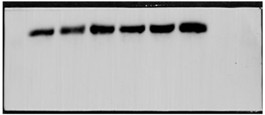

Full and uncropped western blot for Figure 3B

STAT3

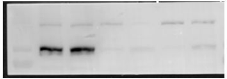

ACTB

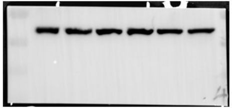

Full and uncropped western blot for Figure 4A

TRIM21

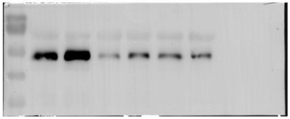

ACTB

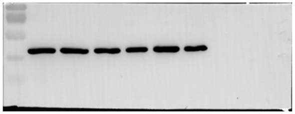

Full and uncropped western blot for Figure 4E

TRIM22

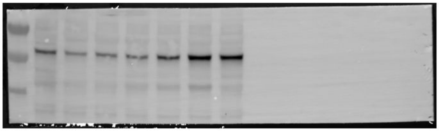

ACTB

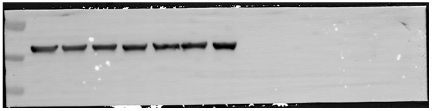

Full and uncropped western blot for Figure 4F

TRIM22

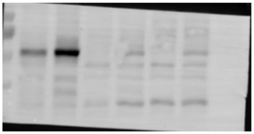

ACTB

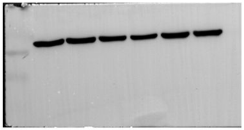

Full and uncropped western blot for Figure 4J

TRIM22

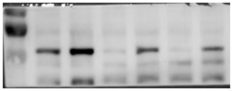

SLC7A11

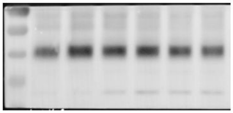

HMOX1

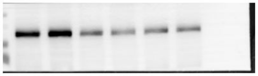

FSP1

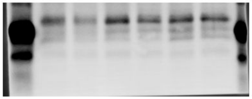

GPX4

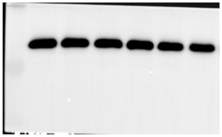

ACTB

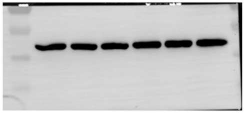

Full and uncropped western blot for Figure 5F

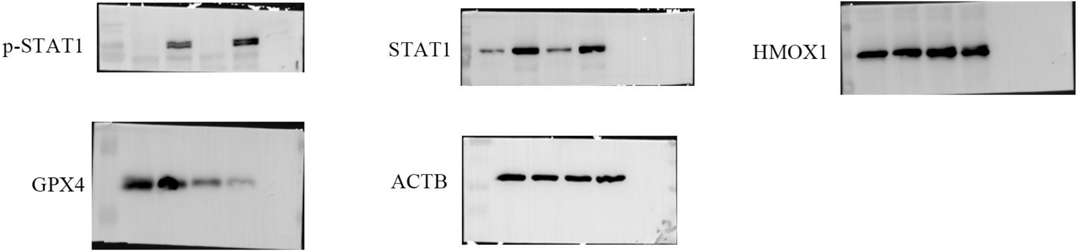

Full and uncropped western blot for Figure S3C

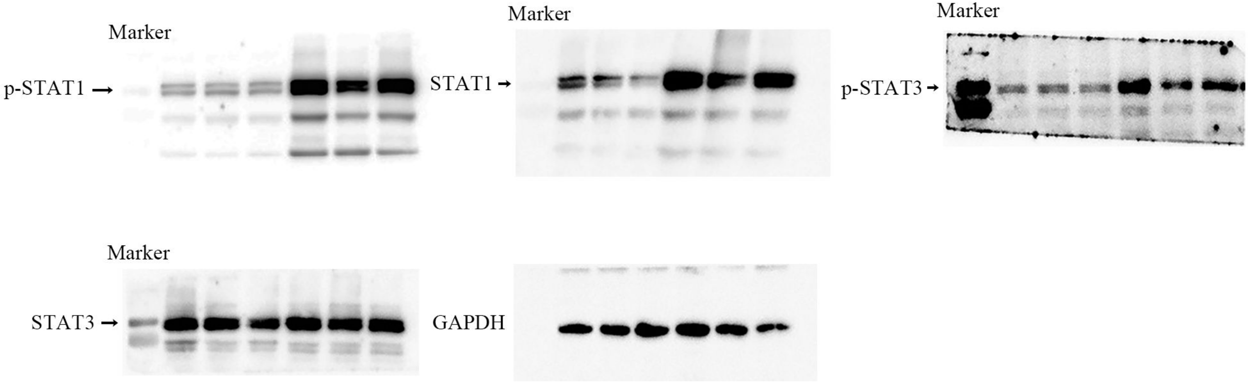

Full and uncropped western blot for Figure S5D

p-STAT1

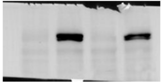

STAT1

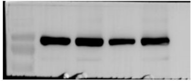

GPX4

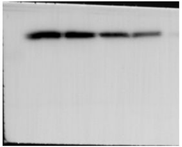

HMOX1

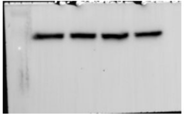

ACTB

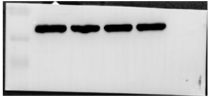

Supplement: Supplementary file 3 [file DataSheet3.pdf]
